# Trends and patterns of incidence of diffuse glioma in adults in the United States, 1973‐2014

**DOI:** 10.1002/cam4.1757

**Published:** 2018-09-02

**Authors:** Kai Li, Dan Lu, Yazhou Guo, Changwei Wang, Xiao Liu, Yu Liu, Dezhong Liu

**Affiliations:** ^1^ Department of Neurosurgery Zhoukou Central Hospital Zhoukou Henan Province China; ^2^ Medical Examination Center Zhoukou Central Hospital Zhoukou Henan Province China

**Keywords:** age‐period‐cohort model, astrocytoma, cancer incidence, glioblastoma, trend

## Abstract

**Introduction:**

The objective of the study was to identify trends in incidence of adult diffuse gliomas in the United States and evaluate the contribution of age, period, and cohort effects to the trends.

**Methods:**

Using the Surveillance, Epidemiology, and End Results 9 database, primary diffuse glioma patients (≥20 years old) diagnosed from 1973 to 2014 were identified. Incidence trends were analyzed using joinpoint regression and age‐period‐cohort modeling.

**Results:**

Overall, the incidence for adult glioma decreased slowly from 1985 to 2014 (annual percent change [APC] = 0.5%, 95% confidence intervals [CI], 0.3%‐0.6%). In histology subtype‐stratified analysis, glioblastoma and nonglioblastoma exhibited opposite trends. The incidence for glioblastoma increased from 1978 to 2014 (APC for year 1978‐1992 = 2.7%, 95% CI, 1.8%‐3.6%; APC for 1992‐2014 = 0.3%, 95% CI, 0%‐0.6%), while the incidence for nonglioblastoma decreased significantly from 1982 to 2014 (APC = 2.2%, 95% CI, 2.0%‐2.5%). Age‐period‐cohort modeling revealed significant period and cohort effects, with the patterns for glioblastoma and nonglioblastoma distinctive from each other. Compared with adults born 1890s, those born 1920s had approximately 4‐fold the risk of glioblastoma after adjustment of age and period effects, while the risk of nonglioblastoma was reduced by half in individuals in the 1939 cohort as compared with those in the 1909 cohort.

**Conclusions:**

The results support the hypothesis of etiological heterogeneity of diffuse gliomas by histology subtypes. The established risk factors cannot fully explain the distinct patterns by histology subtypes, which necessitate further epidemiological studies.

## 1 INTRODUCTION

According to estimates for 2017, 13 450 men and 10 350 women in the United States will be diagnosed with primary malignant tumors of the brain and other parts of the central nervous system (CNS), accounting for 1.4% of all cancer diagnoses this year.^1^ Approximately 75% of primary brain and other CNS cancers are gliomas originated from glial or precursor cells, the vast majority of which are diffuse gliomas.^2^ Diffuse gliomas, often characterized by extensive infiltrative growth of glial cells in the CNS parenchyma, are traditionally classified based on histology as astrocytoma, oligodendroglioma, or mixed oligoastrocytoma, and graded as WHO grade II (low‐grade), III (anaplastic), or IV (glioblastoma).^3^ Glioblastoma (grade IV astrocytoma) is the most aggressive form of brain and other CNS cancers and compromises 55% of gliomas diagnosed in the United States.^2^

Although the overall incidence of invasive cancer of the brain and other CNS has been declining slightly by 0.2% per year over the last 10 years in the United States,^4^ there are considerable variations in incidence trends between different age groups and histology subtypes, such as an increasing trend of pediatric glioma from 2000 to 2013, a decreasing trend of glioma in elder adults from 2007 to 2013, and a decreasing trend of malignant meningioma from 2000 to 2013.^2^ Some ascribed these variations to changes in the brain tumor classification system,^2^ improved detection due to introduction of diagnostic imaging,^5^, ^6^ and changes in awareness and attitude toward diagnosis in elder patients in primary care.^7^, ^8^ Other evidence, however, suggested that these factors may not be as strong as assumed and the explanation is probably multifactorial, including changes in etiology leading to true changes in incidence.^9^, ^10^ Overall, little is known about the etiology of brain tumors. Exposure to therapeutic or high‐dose ionizing radiation, particularly early in life, is the only well‐established environmental risk factor for glioma,^11^, ^12^, ^13^, ^14^ and inherent genetic factors likely exert an effect on cancer occurrence.^15^, ^16^, ^17^ Studies of potential brain tumor risks associated with exposure to nonionizing radiation from cellular phone use produced mixed and inconclusive results.^18^, ^19^ Allergies or atopic diseases (eg, eczema, psoriasis, asthma, and hay fever) have been consistently reported to provide a protective effect against glioma with the probable mechanism linked to enhanced immunesurveillance.^20^, ^21^, ^22^

To better understanding the evolution of adult diffuse gliomas, this study described the patterns and trends in incidence of the disease using the Surveillance, Epidemiology, and End Results (SEER) database. The SEER database provides large population‐based sources of data at the US national level, which is particularly valuable given that gliomas are a relatively rare disease and as such a single clinical setting unlikely accumulates enough cases for comprehensive and accurate estimate. Temporal variations in incidence were compared by glioma histology subtypes and assessed simultaneously by age, calendar period, and year of birth using an age‐period‐cohort model aiming at obtaining a better understanding of possible determinants of the disease trend and etiology of the disease.

## 2 MATERIALS AND METHODS

### 2.1 Data sources and study cohort

Data were obtained from the SEER 9 database, which consists of cancer registries from 9 geographic areas covering about 9.5% of the US population and provides cancer case data from the year 1973 forward.^23^ In this study, all patients age 20 years and older with a first diagnosis of primary diffuse glioma from 1973 to 2014 were eligible for inclusion. Primary diffuse glioma cases were defined with the International Classification of Diseases for Oncology, 3rd Edition (ICD‐O‐3) histology codes 9380, 9382, 9400‐9411, 9420, and 9440‐9460, and were restricted to malignant tumor (behavior code 3) of the brain (ICD‐O‐3 site codes C710‐C719). Histology subtypes were classified into grade II and III astrocytoma (ICD‐O‐3 histology codes 9400‐9411, 9420), oligodendroglioma (9450‐9460), mixed oligoastrocytoma (9382), and glioblastoma (9440‐9442). Furthermore, we grouped astrocytoma, oligodendroglioma and mixed oligoastrocytoma as nonglioblastoma in the comparison analysis against glioblastoma. Cases with histology code 9380 were excluded from analyses stratified by histology subtypes because of the inability to assign these cases to either histology subgroup. The anatomic subsites were classified as supratentorial (ICD‐O‐3 site codes C710‐C714), infratentorial (C716‐C717), and overlapping/not otherwise specified (NOS; C715 and C718‐C719). Demographic data extracted from the SEER 9 database included age at diagnosis, calendar year of diagnosis, sex, and race.

### 2.2 Statistical analysis

Group differences in demographic and clinical characteristics were tested using chi‐square test and post hoc Bonferroni correction for multiple comparisons. Age at diagnosis was categorized as young adult (20‐39 years), middle age (40‐64 years), and elder (≥65 years). Calendar year of diagnosis was categorized into 4 groups: 1973‐1982, 1983‐1992, 1993‐2002, and 2003‐2014. Cochran‐Armitage test for trend was used to assess trends in sex ratio by year of diagnosis. All tests were two‐tailed and statistical significance was assigned *P *<* *0.05. The analyses were carried out using SAS (version 9.1, SAS Institute Inc., Cary, NC, USA).

SEER*Stat program (version 8.3.4, National Cancer Institute, Bethesda, MA, USA) was used to calculate ASRs and corresponding 95% CIs adjusted to the 2000 US standard population by the direct method.^23^ ASRs were stratified by sex (male, female), age (5‐year age interval, 20‐24, 25‐29…80‐84, 85+ years), race (White, Black, and others), histology subtype, and anatomic subsite. Temporal trends of ASRs were quantified with APC using Joinpoint Regression program (version 4.5.0), with up to five joinpoints allowed over the diagnosis years 1973‐2014.^24^ The joinpoint analysis applies permutation test for weighted log‐linear regression to fit linear segments and identify time points at which a significant change in trend occurs. Ten‐year average APC (AAPC) was calculated as geometrically weighted APCs from 2005 to 2014. When describing temporal trends, the terms “increase” or “decrease” were used when the corresponding APC/AAPC was statistically significant (2‐sided *P *<* *0.05); otherwise the terms “stable,” “non‐significant increase,” or “non‐significant decrease” were used.

The age‐period‐cohort model was fitted to the observed crude incidence rate data from the SEER 9 registries using the NCI's online age‐period‐cohort analysis tool.^25^ The model provides parameters that characterize the effects of age, period (year of diagnosis), and cohort (year of birth) related to the observed variations in incidence over time.^26^ Case and population counts were grouped into 14 5‐year age groups (20‐24, 25‐29…80‐84, 85+ years) and eight 5‐year calendar period (1974‐1978, 1979‐1983…2009‐2013), spanning 21 partially overlapping 10‐year birth cohorts referred to by mid‐year of birth (1889, 1894…1989). The model parameters and functions presented in the study include net drift, local drift, longitudinal age curve, period RR, and cohort RR. Net drift evaluates the overall trend of age‐adjusted incidences by period and birth cohort and is analogous to the AAPC in ASRs across the observed period. Local drift evaluates the age‐specific trend of incidences by period and birth cohort. Longitudinal age curve summarizes the age‐specific and cohort‐specific incidences adjusted for period effect and is generally considered superior to cross‐sectional age curve for evaluating the age effects. Period effects were expressed as period RR for each calendar period relative to an arbitrary reference period (herein 1989‐1993), adjusted for age and cohort effects. Cohort effects were expressed as cohort RR for each birth cohort relative to an arbitrary reference cohort (herein the 1939 cohort), adjusted for age and period effects.

## 3 RESULTS

A total of 49 124 patients age 20 years and older diagnosed with a first primary diffuse glioma in the brain during 1973‐2014 were registered in the SEER 9 database. Of these, 3174 (6.5%) patients registered with histology code 9380 (glioma, not otherwise specified) were excluded from subsequent comparison analysis by histology subtypes. A significant trend was observed toward a decreasing proportion of patients with histology code 9380 over time (*P*_trend_ < 0.01, 7.9% in 1973‐1982, 7.2% in 1983‐1992, 5.8% in 1993‐2014). Glioblastoma (58.7%) was the most common subtype, followed by astrocytoma (24.7%), oligodendroglioma (7.3%), and mixed oligoastrocytoma (2.8%). Demographic and clinical features of these patients are summarized in Table 1. Male‐to‐female ratios of approximately 1.3:1 were observed in all histology subgroups with minor fluctuations over time (*P*_trend_ = 0.428). As such, to improve statistical power, data were not stratified by sex in subsequent analyses. Whites made up approximately 90% of the patients regardless histology subtypes. The majority of the primary tumor site was located in the supratentorial brain region, and it was more predominant in oligodendroglioma and mixed oligoastrocytoma.

**Table 1:** Demographic and clinical characteristics of adult patients diagnosed with primary brain glioma: SEER 9, 1973‐2014

	Total (n = 49 124)	Astrocytoma (n = 12 149)	Oligodendroglioma (n = 3608)	Mixed oligoastrocytoma (n = 1358)	Glioblastoma (n = 28 835)	*P* value
Sex (%)						
Male	56.7	55.9	57.3	56.6	57.4	0.056
Female	43.3	44.1	42.7	43.4	42.6	
Age at diagnosis, years (%)						
20‐39	15.5	27.7	38.4	41.2	5.8	<0.001
40‐64	44.8	42.5	48.6	48.1	46.3	
≥65	39.8	29.8	13.0	10.7	47.9	
Race (%)						
White	90.7	90.4	89.2	89.6	91.4	<0.001
Black	4.9	5.2	4.3	4.2	4.7	
Other	4.4	4.5	6.5	6.2	3.9	
Year of diagnosis (%)						
1973‐1982	16.9	24.2	8.0	7.7	14.9	<0.001
1983‐1992	22.9	33.1	15.0	19.4	19.4	
1993‐2002	25.4	20.7	38.6	27.0	25.9	
2003‐2014	34.9	22.0	38.3	45.9	39.8	
Anatomic location (%)						
Supratentorial	70.7	67.7	82.2	82.4	72.0	<0.001
Infratentorial	3.0	5.1	1.0	1.2	1.1	
Overlapping/NOS	26.3	27.2	16.9	16.4	26.9	

NOS, not otherwise specified.

**Figure 1:**
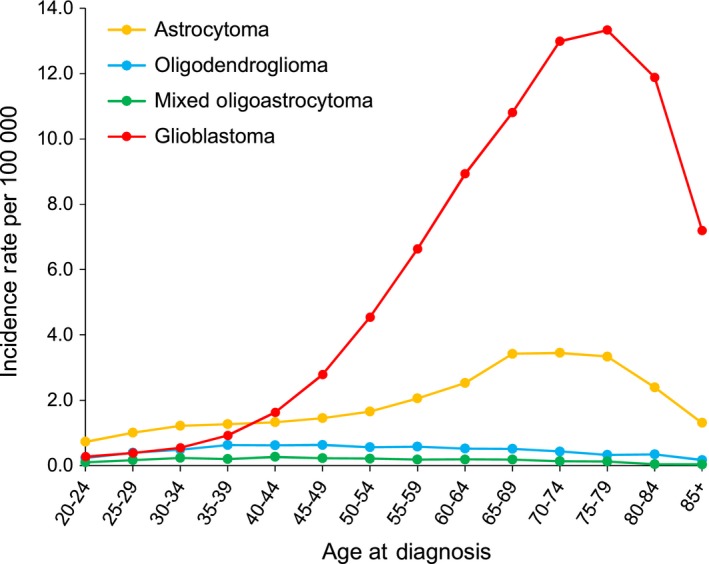
Age‐specific incidence rate for diffuse gliomas by histology subtypes, United States, 1973‐2014. Age‐specific incidence rates were estimated from SEER 9 registries

Age‐specific incidence curves by histology subtypes are displayed in Figure 1. Glioblastoma incidence increased continually with age, reaching a peak at 75‐79 years, and decreased thereafter. Overall, 47.9% patients with glioblastoma were diagnosed at 65 years and older (Table 1). Compared with glioblastoma, the increase in incidence by age was more moderate for astrocytoma, with a gradual rise resulting in a flattened peak at 65‐79 years. Oligodendroglioma and mixed oligoastrocytoma exhibited similar age‐specific incidence curves; the incidence increased sharply until 35‐39 years for oligodendroglioma and 30‐34 years for mixed oligoastrocytoma, then flattened until 45‐49 years, and decreased with advancing age thereafter. Young adults (20‐39 years) made up 38.4% and 41.2% of patients with oligodendroglioma and mixed oligoastrocytoma, respectively, significantly higher than the proportion in patients with glioblastoma (5.8%; *P *<* *0.001 for both; Table 1).

**Figure 2:**
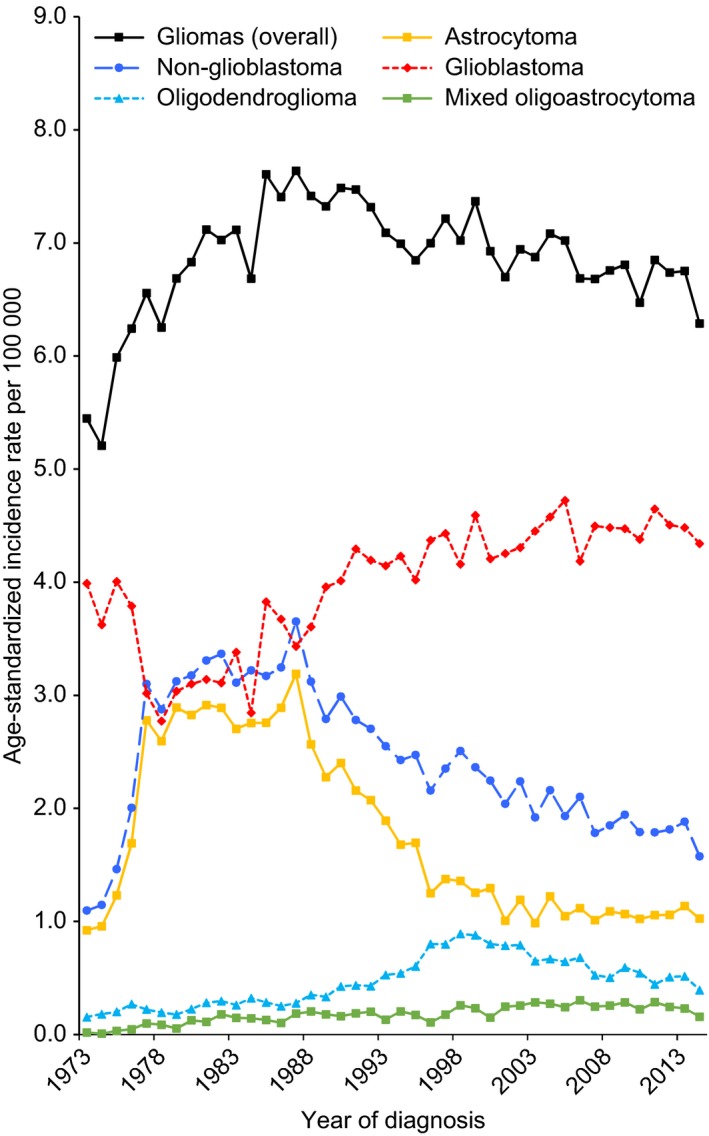
Trends in age‐standardized incidence rate for diffuse gliomas, overall and by histology subtypes, United States, 1973‐2014. Incidence rates estimated from SEER 9 registries were age adjusted to the 2000 US standard population

Annual age‐standardized incidence rates (ASRs; per 100 000 population) between 1973 and 2014 were 6.9 for glioma overall, 4.1 for glioblastoma, 1.7 for astrocytoma, 0.5 for oligodendroglioma, and 0.2 for mixed oligoastrocytoma. ASR curves by histology subtypes and year of diagnosis are illustrated in Figure 2. The incidence for glioma overall increased by 2.4% (95% confidence interval [CI], 1.7%‐3.1%) per year from 1973 to 1985 and then decreased by 0.5% (95% CI, 0.3%‐0.6%) per year from 1985 to 2014 (Table 2). Analysis by histology subtypes revealed that glioblastoma and nonglioblastoma exhibited opposite temporal trends in ASR. The incidence for glioblastoma decreased sharply by 7.3% (95% CI, 3.3%‐11.0%) per year from 1973 to 1978, then increased obviously by 2.7% (95% CI, 1.8%‐3.6%) per year from 1978 to 1992 and the increase slowed to 0.3% (95% CI, 0%‐0.6%) per year from 1992 to 2014. The incidence for nonglioblastoma, however, showed a nonsignificant increasing tendency from 1977 to 1982 (annual percent change [APC] = 3.6%; 95% CI, −2.5% to 9.9%), followed by a decrease of 2.2% (95% CI, 2.0%‐2.5%) per year from 1982 to 2014 (Table 2). As shown in Figure 2, the change of incidence tendency (joinpoint) appeared in 1987 for astrocytoma, decreasing obviously after that year, while the joinpoint delayed to 1998 for oligodendroglioma.

**Table 2:** Annual percent change in brain glioma incidence rates by histology subtypes: SEER 9, 1973‐2014

	Trend 1			Trend 2			Trend 3			Trend 4			2005‐2014	
	Year	APC	95% CI	Year	APC	95% CI	Year	APC	95% CI	Year	APC	95% CI	AAPC	95% CI
Glioma (overall)	1973‐1985	2.4	1.7, 3.1^a^	1985‐2014	−0.5	−0.6, −0.3^a^							−0.5	−0.6, −0.3^a^
Nonglioblastoma	1973‐1977	33.1	21.0, 46.4^a^	1977‐1982	3.6	−2.5, 9.9	1982‐2014	−2.2	−2.5, −2.0^a^				−2.2	−2.5, −2.0^a^
Astrocytoma	1973‐1977	38.6	27.9, 50.2^a^	1977‐1987	0.7	−0.7, 2.1	1987‐2001	−6.8	−7.6, −5.9^a^	2001‐2014	−0.1	−1.2, 0.9	−0.1	−1.2, 0.9
Oligodendroglioma	1973‐1989	3.9	2.1, 5.7^a^	1989‐1998	11.3	8.3, 14.4^a^	1998‐2014	−4.4	−5.2, −3.6^a^				−4.4	−5.2, −3.6^a^
Mixed oligoastrocytoma	1973‐1980	29.8	3.7, 62.4^a^	1980‐2011	2.6	1.7, 3.4^a^	2011‐2014	−15.9	−34.5, 7.9				−4.0	−11.4, 4.0
Glioblastoma	1973‐1978	−7.3	−11.0, −3.3^a^	1978‐1992	2.7	1.8, 3.6^a^	1992‐2014	0.3	0.0, 0.6^a^				0.3	0.0, 0.6^a^

AAPC, average annual percent change; APC, annual percent change; CI: confidence interval.

a The value is statistically different from zero (2‐sided *P *<* *0.05) based on joinpoint regression analysis.

**Figure 3:**
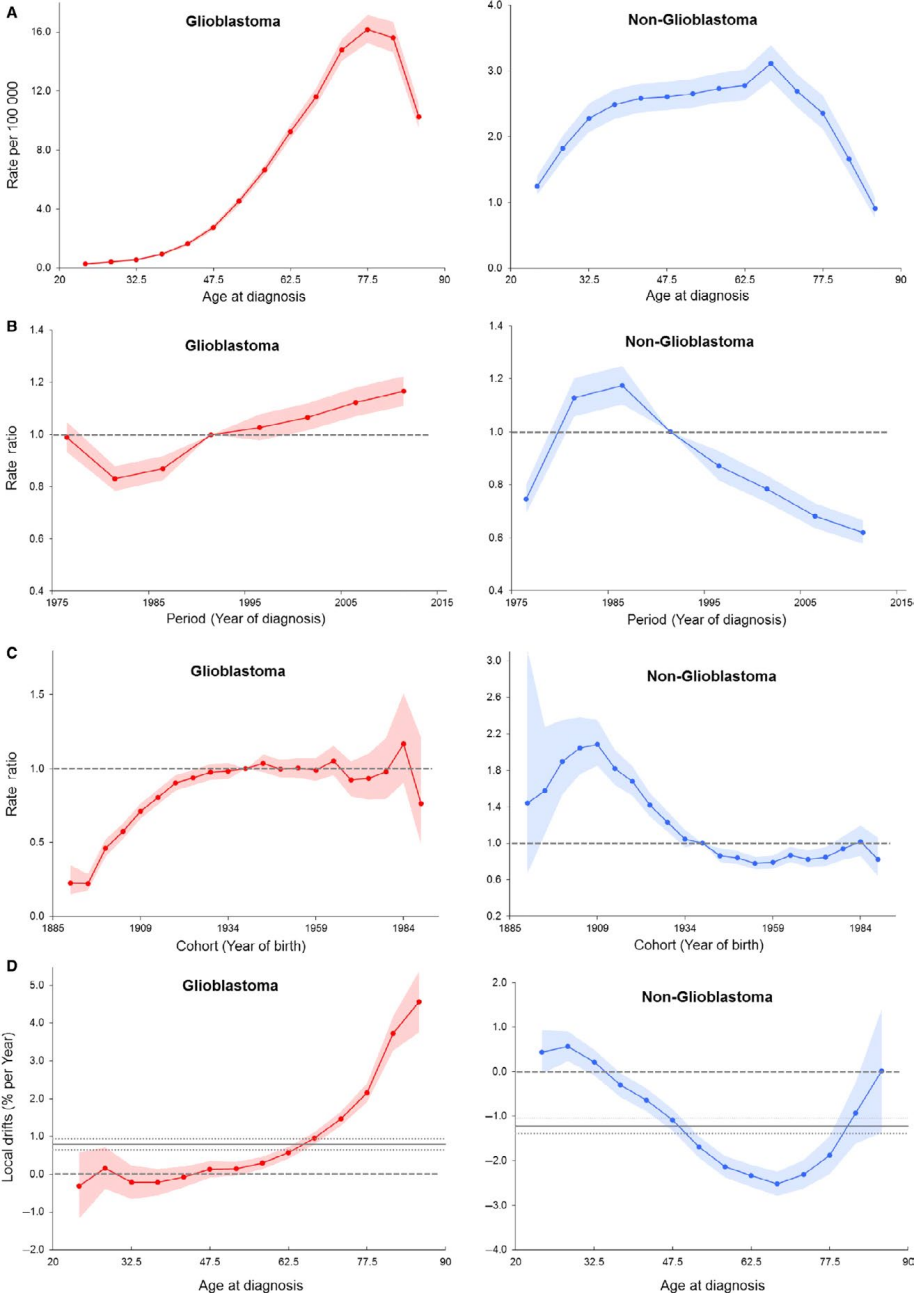
Age‐period‐cohort modeling parameters and functions for incidence for glioblastoma (left) and nonglioblastoma (right). (A) Period rate ratio (B) cohort rate ratio (C) longitudinal age curve, and (D) local drifts with net drift. Shaded bands indicate 95% confidence intervals. Dash lines in (A) and (B) indicate reference period and cohort, respectively. Solid horizontal line and upper and lower dot lines in (D) indicate net drift and its 95% confidence intervals

The age‐period‐cohort modeling fitting indicated both period and cohort effects of the incidence trends of glioblastoma and nonglioblastoma, with period and cohort deviations significantly different from zero (*P *<* *0.001 for all, Wald tests). The period effects of glioblastoma and nonglioblastoma are displayed in Figure 3A. Using 1989‐1993 as the reference period, relative risks of glioblastoma decreased until 1979‐1983 and increased thereafter, whereas for nonglioblastoma, relative risks increased until 1979‐1983 and decreased thereafter, showing an opposite period effect. The cohort effects glioblastoma and nonglioblastoma are displayed in Figure 3B, present as cohort rate ratio (RR) and using the 1939 cohort as the reference group. For glioblastoma, relative risks increased for cohorts born from 1890s until 1920s, then remained relative stable over the cohorts born after 1920s with some fluctuations particularly for the recent cohorts. Compared with the individuals born 1890s, those born 1920s and after had approximately 4‐fold the risk of glioblastoma after adjustment of age and period effects. Relative risks of nonglioblastoma increased for the first several cohorts born from late 1880s until the 1910s, followed by gradual decrease in the subsequent cohorts and minor upwards since the cohort born late 1960s. Specifically, the risk of nonglioblastoma was reduced by half in individuals in the 1939 cohort as compared with those in the 1909 cohort (cohort RR = 0.48, 95% CI, 0.42‐0.54).

The longitudinal age curves of glioblastoma and nonglioblastoma incidence are displayed in Figure 3C. The risk of glioblastoma monotonically increased with age until peaking at 75‐79 years and decreased thereafter. Compared with glioblastoma, although different in the shape of up‐slope, nonglioblastoma had a similar overall age curve pattern, with the risk increasing with age until peaking at 65‐69 years and decreasing thereafter.

The local drift values, which indicate the age‐specific APCs within the observed period, are present in Figure 3D. All local drift values were above 0 in adults age 55 years and older for glioblastoma and were higher with advanced age, with the peak value of 4.57% per year in adults age 85 years and older (95% CI, 3.76%‐5.38%). On the other hand, most local drift values were below 0 for nonglioblastoma, reaching the lowest in adults age 60‐64 years (percent per year = −2.34%, 95% CI, −2.59% to −2.09%). As references, the net drift values, which indicate the overall APC within the observed period, were 0.79 (95% CI, 0.64‐0.93) and −1.22 (95% CI, −1.39 to −1.05) for glioblastoma and nonglioblastoma, respectively.

## 4 DISCUSSION

Using data from US SEER registries, we confirmed that while the overall incidence of adult gliomas has been slowly decreasing since mid‐1980s, there has been a significant increasing trend in the incidence of glioblastoma coupled with a significant decreasing trend of nonglioblastoma, mostly attributable to grade II and III astrocytoma. We complemented standard descriptive analyses with an age‐period‐cohort modeling approach, both of which indicated distinct temporal patterns and age‐related differences in incidence of adult diffuse gliomas by histology subtypes in the US population. Age‐period‐cohort modeling revealed strong and significant period and birth cohort effects on the observed incidence trends of glioblastoma and nonglioblastoma, and furthermore, their birth cohort and period effect patterns were distinct from each other, supporting the hypothesis of etiological heterogeneity of diffuse gliomas by histology subtypes.

Our results of rising trend of glioblastoma incidence and trend variations by glioma histology subtypes are in line with previous findings in the United States and European countries.^27^, ^28^, ^29^, ^30^ The observed distinct period and birth cohort effect patterns of glioblastoma and nonglioblastoma could be due to a number of histology‐specific factors that dynamically affect the population. However, these histology‐specific factors are largely unknown or postulated. It is known that incidence is greatly influenced by local cancer registry diagnosis coding. SEER uses the WHO classification of CNS tumors for classifying brain tumor subtypes, which has been revised four times since first published in 1979, each time with considerable changes. These changes may have partially contributed to the temporal trends of gliomas but how these changes have affected histology‐specific incidences remains unclear.

Period effects are generally interpreted as variation in risk due to population‐wide environmental changes and/or changes in diagnostic criteria and disease classification that affect all age groups equally.^26^, ^31^ In the present study, we found that the risk of glioblastoma increased since the period of 1979‐1983, and meanwhile the risk of nonglioblastoma decreased since the period of 1984‐1988. Since its wide availability in late 1970s, CT utilization has been steadily increasing in the United States and thus might contribute to increased detection/improved diagnosis of glioblastoma.^32^ A previous study using SEER‐Medicare database supported this interpretation, but limited to elderly patients due to data availability, and attributed the increasing use of CT scan to a more aggressive pursue of diagnosis by physicians.^10^ Ionizing radiation exposure from CT scan might also contribute to the positive period effect for glioblastoma, given that epidemiological studies provided inconclusive but promising results regarding the association between CT scan and increased glioma risk.^12^, ^33^ On the other hand, MRI is sensitive to low‐grade glioma and has been linked to improved detections of childhood brain tumors since its introduction in mid‐1980s.^5^, ^34^ However, despite increased use of MRI, a negative period effect for nonglioblastoma since the year 1984 was observed in this study, suggesting that the influence of MRI to improved diagnosis of nonglioblastoma in adult was not significant or was offset by other factors that are largely unknown.

Birth cohort effects are generally interpreted as variation in risk due to changes in the exposure to risk factors affecting age groups unequally, which could be due to unequal distribution of the exposure in population or unequal effect of a population‐level exposure to different age groups who are at a critical development period.^31^ The strong birth cohort effects observed in this study likely arise from changes in environmental and lifestyle factors that occur relatively early in life. Exposure to ionizing radiation is the established environmental factor associated with increased risk for gliomas and children are more susceptible to its oncogenic effect. It has been reported that the annual radiation dose per individual has increased significantly in the United States over the past decades, largely due to the growth of medical imaging procedures.^32^, ^35^, ^36^ However, this study could not ascertain the contributing role of radiation exposure to the rising incidence of glioblastoma due to the limitation of study design. The rapid expansion of cellular phone use has brought concern regarding influence over glioma, with some evidence suggesting significant association between cellular phone use, particularly long‐term user (≥10 years) and user with first use at early age, and risk of low‐grade glioma.^37^, ^38^ However, other studies have shown no significant relationship or even an inverse relationship between cellular phone use and incidence trends of glioma.^39^, ^40^, ^41^, ^42^ Although allergy history is thought to be protective against risk for glioma, antihistamine use was associated with increased risk for grade III astrocytoma, while nonsteroidal anti‐inflammatory drug use was associated with reduced risk for glioblastoma.^43^ Thus, it is possible that use of these medications might partly explain the difference in birth cohort patterns of glioblastoma and nonglioblastoma. Obesity epidemic is parallel with the increase in glioblastoma incidence. Although data are limited, it appears that energy balance in early life was related to glioma risk, as evidenced by significantly increased risk for glioma among individuals who were tall, obese at age 18, and inactive during adolescence.^44^, ^45^ On the other hand, obesity in adults seems to be unrelated to glioma risk.^45^ These studies, however, did not provide subgroup analysis results by glioma subtypes. Given the long latency period between the exposure to these potential environmental and lifestyle factors and onset of glioma, it is extremely difficult to identify the determining factors for the birth cohort effects. Moreover, genetic factors likely complicate their effects on disease development.

Our findings of differential longitudinal age curve and local drifts support an obvious difference in age‐related natural history in adult diffuse gliomas by histology subtypes, and the etiological heterogeneity, particularly genetic variations, likely contribute to this age‐related difference.^46^, ^47^ Although similar age difference by histological subtypes has been reported in previous studies, results may be limited due to not adjusting for cohort and/or period effects.^48^

Primary glioblastoma constitutes the majority of glioblastoma cases (~95%) and is considered to arise rapidly de novo (ie, no evidence of a low‐grade precursor lesion), whereas secondary glioblastoma develops through progression from grade II or III astrocytoma and mostly affects younger adults (mean age of 45 years).^49^ In the present study, about half of glioblastoma patients were elderly (≥65 years), consistent with the age distribution of primary glioblastoma.^49^

This study is subject to several limitations. First, the histologic criteria for CNS tumor have been revised several times in history, which likely contribute to temporal trends of histological subtypes. Thus, we could not exclude the possibility that variances in subtypes, particularly those nonglioblastoma ubtypes, may be due to changes in CNS tumor classification instead of real change in disease incidence. Second, while SEER registries maintain high standards for data quality, given that classification of gliomas often depends on considerable subjective judgment and is changing overtime, the possibility of misclassification could not be excluded. In this study, for APC modeling, we adopted the categorization used in a previous study,^48^ grouping gliomas into glioblastoma and nonglioblastoma in consideration of the accuracy of diagnosis. This approach, however, was unable to distinguish possible age, period, and cohort effects specific to each of the histological subtypes. Third, longitudinal data on potential risk factors were not available in SEER databases, which limited the study's ability to evaluate their impacts on temporal trends of gliomas. Fourth, while SEER registries achieve very high completeness in case ascertainment, SEER 9 only represents about 9.5% of the US population, and thus the temporal trend patterns observed in the study may not be generalizable to the national population. No obvious difference was observed regarding the glioma incidence rates of year 2000‐2014 between SEER 9 and SEER 18, the latter covering 28% of the US population (data not shown). Fifth, no genetic profile was provided in SEER database to distinguish primary from secondary glioblastoma cases. Finally, although the age‐period‐cohort modeling could provide etiological clues related to the temporal trends, no direct evidence was generated from the modeling. Furthermore, the etiological interpretations should be viewed with caution due to the limitations inherent to the age‐period‐cohort modeling, such as collinearity among age, period and cohort.^26^

In summary, our study showed that although the incidences of glioblastoma, grade II and III astrocytoma, oligodendroglioma, and mixed oligoastrocytoma changed at different degrees from 1973‐2014, the overall incidence of diffuse gliomas remained relatively stable over the last decades largely because these changes offset each other. The distinct patterns of age, period, and birth cohort effects between glioblastoma and nonglioblastoma support etiological heterogeneity and may partly due to changes in classification system and improved diagnostic imaging. The established risk factors for gliomas, however, cannot fully explain the trend difference by histology subtypes, which necessitates more epidemiological studies to identify the underlying causes of for the distinction.

## CONFLICT OF INTEREST

The authors have declared that no competing interest exists.

## ETHICAL APPROVAL

All procedures performed in studies involving human participants were in accordance with the ethical standards of the institutional and/or national research committee and with the 1964 Helsinki declaration and its later amendments or comparable ethical standards. For this type of study formal consent is not required.
